# Cereal Vinegar Sediment Modulates the Gut Microbiota–Metabolite Axis Associated with Hyperlipidemia in *Apoe*^−/−^ Mice

**DOI:** 10.3390/foods15030427

**Published:** 2026-01-24

**Authors:** Wenhui Duan, Qijie Guan, Yilin Ren, Jin-Song Shi, Zheng-Hong Xu, Yingyue Sheng, Yuzheng Xue, Chengcheng Zhang, Yan Geng

**Affiliations:** 1 Key Laboratory of Carbohydrate Chemistry and Biotechnology, Ministry of Education, State Key Laboratory of Food Science and Resources, School of Food Science and Technology, Jiangnan University, Wuxi 214122, China; duanwhyy@163.com; 2 Department of Gastroenterology, Affiliated Hospital of Jiangnan University, Wuxi 214122, China; renyilin@jiangnan.edu.cn (Y.R.); mini_rae@163.com (Y.S.); yuzhengxue_ahjnu@126.com (Y.X.); 3 Department of Microbial Pathogenesis, School of Medicine, Yale University, New Haven, CT 06519, USA; qijie.guan@yale.edu; 4 School of Life Sciences and Health Engineering, Jiangnan University, Wuxi 214122, China; shijs@163.com; 5 College of Biomass Science and Engineering, Sichuan University, Chengdu 610065, China; zhenghxu@scu.edu.cn

**Keywords:** cereal vinegar sediment, gut microbiota, metabolomic profiles, metabolome, hyperlipidemia

## Abstract

Cereal vinegar sediment (CVS), a byproduct of traditional vinegar fermentation, has been regarded as a health-promoting product. However, its role in genetically induced hyperlipidemia remains unclear. This study systematically evaluated the effects of Dade-CVS (DD-CVS) and Hengshun-CVS (HS-CVS) on apolipoprotein-E-deficient (*Apoe*^−/−^) mice. Both CVS varieties significantly improve certain serological parameters of *Apoe*^−/−^ mice, although the overall impact on serum indicators remains limited. Nevertheless, 16S rRNA sequencing revealed that CVS treatment reshaped gut microbial communities to a notable extent. Compared with the *Apoe*^−/−^ mice, the DD-CVS treatment significantly increased the relative abundance of *Dubosiella* while reducing the genus *Desulfovibrio*, whereas the HS-CVS treatment inhibited the growth of *Bifidobacterium* and *Akkermansia*. The pathways predicted in the KO-DD group included vitamin, amino acid, and energy metabolism, while HS-CVS treatment was associated with bile acid biosynthesis and energy pathways. Metabolomic analysis showed that several key metabolites, including N1-acetylspermidine, succinic acid, and 25-hydroxycholesterol, were significantly altered following CVS supplementation. Correlation analysis revealed significant associations between serum indicators and these metabolites. *Alistipes*, *Enterorhabdus*, and *Romboutsia* were also correlated with serum indicators. Overall, these findings indicate that CVS primarily modulated the gut microbiota–metabolite axis and partial lipid modulation in hyperlipidemic mice. The study provides a reference for studies on the beneficial functions of CVS in hyperlipidemia.

## 1. Introduction

Cereal vinegar sediment (CVS) is a byproduct of traditional Chinese vinegar fermentation, produced during the aging process of vinegar. Previous studies have demonstrated that CVS is enriched with nutrients and bioactive compounds such as sugars, proteins, polyphenols, and flavonoids. Due to these constituents, CVS has traditionally been utilized in Chinese medicine and is reported to exert pharmacological effects against enteritis, hyperlipidemia, hyperglycemia, hepatoprotective, senile dementia, and ulcerative colitis in mice [1,2,3,4,5]. Vinegar sediment has also been shown to exert beneficial effects on the intestinal tract. A study reported that Kurozu Moromi paste, a Japanese black vinegar sediment, suppressed the development of colon cancer in mice, whereas vinegar alone did not exhibit such inhibitory effects on tumor growth [6]. Geng et al. have reported that CVS regulated the gut microbiota and alleviated spontaneous ulcerative colitis in *Il-10*-deficient mice [5]. Meanwhile, studies demonstrate that CVS exerts hepatoprotective effects and modulates the gut microbiota in mice [1,7].

In recent decades, metabolic disorders such as obesity, diabetes, and atherosclerosis have become increasingly prevalent alongside improvements in living standards [8]. Notably, dyslipidemia, or hyperlipidemia, is a common clinical feature of these chronic conditions. The etiology of hyperlipidemia involves both genetic and environmental factors, including diet and lifestyle. However, the underlying mechanisms remain incompletely understood. Growing evidence indicates that the gut microbiota and host metabolism play crucial roles in the development of hyperlipidemia [9]. Individuals with hyperlipidemia have been shown to exhibit both structural and functional alterations in the gut microbial community [10]. Moreover, gut microbiota–derived metabolites serve as important regulators of lipid homeostasis [9]. For example, berberine has been reported to exert lipid-lowering effects by promoting intestinal butyrate production, inhibiting bile salt hydrolase activity in the gut microbiota, and modulating farnesoid X receptor (FXR) signaling in the ileum [11].

Diet is an important factor affecting gut microbiota and metabolism [12]. Evidence from animal studies demonstrates that a diet rich in fermented foods is more likely to enhance gut microbiota homeostasis and host health [13,14]. CVS has been reported as a by-product of vinegar aging with notable pharmacological activities in mice. However, it remains unclear whether and how CVS intervention modulates gut microbial homeostasis and metabolic profile in hyperlipidemic mice. This study’s primary focus is to determine whether and how Dade-cereal vinegar sediment (DD-CVS) and Hengshun-cereal vinegar sediment (HS-CVS) modulate the gut microbiota and its metabolic profile, with serum lipid markers evaluated as secondary host response indices in hyperlipidemic mice. The study provides a reference for research on the health functions of CVS in hyperlipidemia.

## 2. Materials and Methods

### 2.1. Materials and Reagents

CVS was provided by the laboratory (Wuxi, China). Aged vinegar and its natural precipitate were centrifuged at 7500× *g* for 20 min. After centrifugation, the precipitate was collected as CVS. The basic compositional profiles of the two CVS types are provided in Table S1 of the Supplementary Materials. Acetonitrile, methanol HPLC-MS-grade methanol, and anhydrous methanol for non-targeted metabolomics were obtained from Fisher Scientific (Waltham, MA, USA).

### 2.2. Animals and Treatments

**Figure 1:**
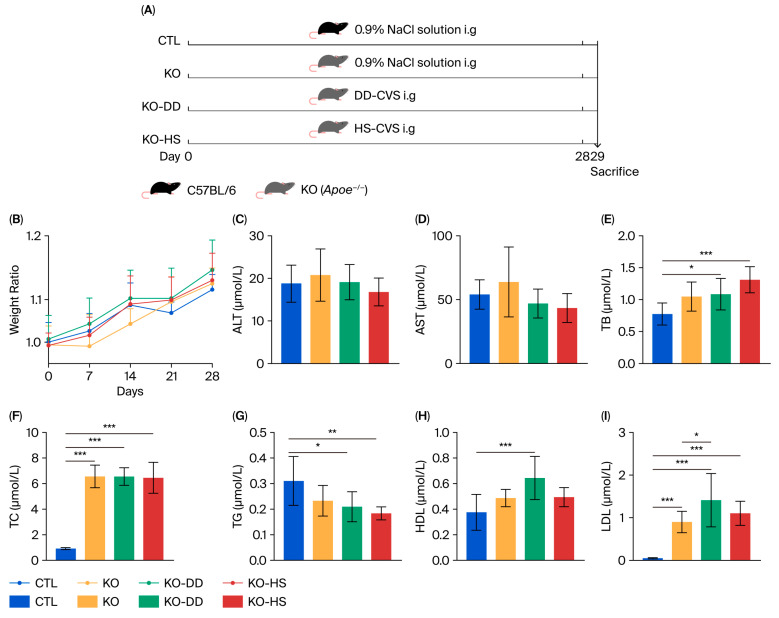
The effects of CVS treatment on *Apoe*^−/−^ mice. (**A**) The animal treatment process. (**B**) The body weight of mice. The levels of ALT (**C**), AST (**D**), TB (**E**), TC (**F**), TG (**G**), HDL (**H**), LDL (**I**) in serum. Data presented as mean ± SD (*n* = 7–8). * *p* < 0.05, significant difference; ** *p* < 0.01, highly significant difference; *** *p* < 0.001 extremely significant difference.

Apolipoprotein E-deficient (*Apoe*^−/−^) mice exhibit marked symptoms of hyperlipidemia and are internationally recognized models of dyslipidemia [15,16]. C57BL/6J mice (male, 5 weeks old) and B6/JGpt-Apoe*em1Cd82*/Gpt mice (male, 5 weeks old) were purchased from GemPharmatech Co., Ltd. (Nanjing, China). The experimental unit was a single mouse, with 8 mice in each group. In total, 32 mice were used in the entire experiment. The sample size was determined based on previous similar studies and a statistical power analysis to ensure an 80% power at a significance level of α = 0.05 to detect the expected effect. Mice had free access to water and food for a week. Mice were maintained under controlled conditions consisting of a 12 h light/dark cycle, a temperature of 22 ± 2 °C, a humidity of 55 ± 5%, and a noise level of ≤60 dB. After the acclimation period, the wild-type C57BL/6J mice were defined as the control (CTL) group (*n* = 8). The *Apoe*^−/−^ mice were randomly and equally divided into three groups, including the *Apoe*^−/−^ (KO) group (*n* = 8), the Hengshun-cereal vinegar sediment treatment (KO-HS) group (*n* = 8), and the Dade-cereal vinegar sediment treatment (KO-DD) group (*n* = 8). The animal treatment process is shown in Figure 1A. The KO-DD group and KO-HS group were gavaged with CVS (1 g/kg day^−1^ body weight) once a day with standard chow. This dosage was determined by referring to our previous research [5]. The CTL group and KO group were gavaged with the same volume of sterile water with standard chow. The gavage continued for 4 weeks, and the weight changes in the mice were recorded every day. After the experiment, each mouse was euthanized. Blood, tissue, cecal contents, and feces samples were collected for subsequent experiments. All experimental operations were approved by the Experimental Animal Welfare Ethics Committee of Jiangnan University (JN.No20211115m0881228[449]).

Inclusion and exclusion criteria: All mice underwent a health screening before the experiment to ensure they were in good condition. All mice were used in the final analysis.

Excluded data points: Due to insufficient serum samples from one mouse in the CTL, KO, and KO-HS groups, serum assays were excluded as follows: AST, ALT, TC, and TG in the CTL group; AST, ALT, TC, and HDL in the KO group; AST, TG, and ALT in the KO-HS group.

Sample size for each analysis: The sample size for analysis of AST, ALT, TC, and TG in the CTL group was *n* = 7. The sample size for analysis of AST, ALT, TC, and HDL in the KO group was *n* = 7. The sample size for analysis of AST, TG, and ALT in the KO-HS group was *n* = 7. The sample size for the non-targeted metabolomics in the CTL, KO, KO-HS, and KO-DD groups was *n* = 5, 6, 4, and 7, respectively. The sample size for other analyses was *n* = 8.

Randomization of experimental units: Randomization of mice into control and treatment groups was performed with a random number table to guarantee unbiased group assignment.

Strategy to minimize confounders: Mice were handled in a randomized order throughout the experiment, and cage positions were randomly assigned to minimize the influence of environmental factors on the results.

During the experiment, treatment and data analysis personnel were blinded to group allocation, with only the personnel responsible for allocation aware of the group assignments.

### 2.3. Biochemical Examination

Serum aspartate aminotransferase (AST), alanine aminotransferase (ALT), triglyceride (TG), total bilirubin (TB), total cholesterol (TC), high-density lipoprotein (HDL), and low-density lipoprotein (LDL) were detected using an automatic serum biochemical analyzer (ZY310, Shanghai Kehua Bio-Engineering Co., Ltd., Shanghai, China) with the corresponding kits (Nanjing Jiancheng Bioengineering Institute, Nanjing, China).

### 2.4. 16S rRNA Gene Sequencing

DNA was obtained from feces using the DNeasy PowerSoil kit (Mo Bio/QIAGEN, Carlsbad, CA, USA). The bacterial 16S rRNA gene V3~V4 region of the bacterial 16S rRNA gene was amplified using primers 338F (5′-ACTCCTACGGGAGGCAGCAG-3′) and 806R (5′-GGACTACHVGGGTWTCTAAT-3′). PCR was performed in a 25 µL reaction mixture containing 0.2 µM of each primer, 12.5 ng template DNA, and 1× master mix, using the following thermocycling conditions: initial denaturation at 95 °C for 3 min; 30 cycles of denaturation at 95 °C for 30 s, annealing at 55 °C for 30 s, and extension at 72 °C for 30 s; followed by a final extension at 72 °C for 5 min. Amplicon libraries were pooled in equimolar amounts and sequenced on an Illumina NovaSeq 6000 platform (Illumina, San Diego, CA, USA) using paired-end sequencing, with an average sequencing depth of approximately 3.0 × 10^5^ reads per sample.

Raw paired-end reads were demultiplexed and processed using QIIME2 (version 2020.2). Quality filtering and denoising were performed with the DADA2 plugin, which models sequencing errors to infer exact amplicon sequence variants (ASVs) rather than clustering reads into operational taxonomic units (OTUs). ASVs with a total abundance of fewer than 4 reads across all samples or present in fewer than 2 samples were discarded. The resulting ASV abundance table (QIIME2 artifact filtered_feature_table.qza) and corresponding representative sequences were used for all subsequent diversity and taxonomic composition analyses.

For taxonomic annotation, representative ASV sequences were classified with a Naïve Bayes classifier trained on the SILVA reference database (Silva_V138_2019.12.16), trimmed to the V3–V4 region targeted by our primers. Taxonomy was assigned using a confidence threshold of 99%. ASVs that were prevalent in negative controls or classified as mitochondria, chloroplasts, or non-bacterial lineages were removed prior to analysis.

Predicted functional profiles were generated using PICRUSt2 (version 2.3). The ASV representative sequences and their corresponding abundance table were exported from QIIME2 and used as input for the PICRUSt2 pipeline. Briefly, PICRUSt2 places ASVs into a reference phylogeny, normalizes their abundances according to the predicted 16S rRNA gene copy number, and infers KEGG Ortholog (KO), Enzyme Commission (EC), and MetaCyc pathway abundances. The resulting functional profiles were used for downstream differential and pathway enrichment analyses. Functional annotation analysis using PICRUSt2 provided only an approximate, exploratory assessment of potential microbial functions.

### 2.5. Non-Targeted Metabolomics

Fecal samples (~50 mg) were weighed and placed in 2 mL QSP tubes, followed by the addition of 400 μL of pre-chilled methanol with internal standards. The samples were vortexed for 2 min, and centrifuged (15,000× *g* for 5 min at 4 °C) to collect the supernatant for LC–MS analysis. Metabolites were profiled on an AB SCIEX X500R ultra-high-performance liquid chromatography–electrospray ionization–source-time-of-flight mass spectrometry system (UPLC-QTOF, AB SCIEX, Framingham, MA, USA), with electrospray ionization (ESI) in both positive and negative ion modes. Separation was performed on a Thermo Hypersil GOLD column (1.9 μm, 2.1 × 100 mm) maintained at 50 °C, with a 2 μL injection volume and a flow rate of 0.3 mL/min. Mobile phase A was acetonitrile and mobile phase B was 0.1% formic acid in water. The 15 min gradient was: 0–1 min, 95% B; 1–13 min, 95% → 1% B; 13–15 min, 1% B; followed by re-equilibration at 95% B until 18 min.

In ESI+ mode, source parameters were: spray voltage 3500 V, sheath gas 40 arb units, auxiliary gas 10 arb units, sweep gas 1 arb unit, ion transfer temperature 275 °C, and vaporizer/heater temperature 320 °C. Full-scan data were acquired at 60,000 resolution over *m*/*z* 70–1050 (RF lens 70%), followed by ddMS2 with Top5 data-dependent acquisition at 15,000 resolution. In ESI− mode, source parameters were: spray voltage −2800 V, sheath gas 40 arb units, auxiliary gas 10 arb units, sweep gas 1 arb unit, ion transfer temperature 275 °C, and heater temperature 320 °C. Full-scan data were acquired at 60,000 resolution over *m*/*z* 70–1050 (RF lens 70%), followed by ddMS2 with Top5 DDA at 15,000 resolution.

Instrument control and data integration were performed using Analyst Software 1.6.2. The orthogonal partial least-squares discrimination analysis (OPLS-DA) was used to assess the model’s predictive ability, with R2X and Q2 parameters. The model was subjected to permutation testing (n = 200) to check for overfitting, with R2 and Q2 intercepts obtained. One-way Analysis of Variance (ANOVA) was performed to calculate the *p*-value of metabolites, and fold change (FC) was calculated for each CVS treatment group relative to the KO group. A threshold of *p* < 0.05 and FC > 1 was used for preliminary screening of differential metabolites. Metabolomics data were analyzed and plotted using MetaboAnalyst (v 6.0).

### 2.6. Statistical Data

The experimental data were expressed as “mean ± standard”. The differences between the two groups were statistically analyzed by the unpaired *t*-test method. The differences between multiple groups were analyzed using ANOVA with *p* < 0.05 as the standard of significant difference, and the significance was determined by the Tukey test. The data obtained were plotted using GraphPad Prism 9.0 software. The Pearson correlation coefficient was calculated using the R package (v3.6.3).

## 3. Results

### 3.1. Effect of CVS on Apoe^−/−^ Mice

*Apoe*^−/−^ mice were employed as the experimental model (Figure 1A). After 28 days of DD-CVS and HS-CVS administration, all mouse groups showed similar body weight growth (Figure 1B). Analysis of serological indicators revealed that AST and ALT levels were slightly elevated in the KO group, though not significantly, and CVS treatment only marginally reduced these levels (Figure 1C,D). Levels of TB and TC, hallmark indicators of hyperlipidemia, were significantly increased in the KO group compared with the CTL group, and these increases were not reversed by CVS treatment (Figure 1E,F). In contrast, TG levels were significantly reduced following treatment with both CVS and were lower than those in the KO group (Figure 1G). As a protective serum protein with anti-atherosclerotic function, HDL reached its highest level in the KO-DD group, being significantly higher than in the normal group and higher, though not significantly, than in the model group (Figure 1H). The level of LDL significantly increased in the KO group compared with the CTL group, but changes in the CVS treatment groups were not significant (Figure 1I). This indicates that CVS significantly improves certain serological parameters in *Apoe*^−/−^ mice, although its overall impact on serum indicators in hyperlipidemic mice remains limited.

### 3.2. Effect of CVS on Gut Microbiota in Apoe^−/−^ Mice

**Figure 2:**
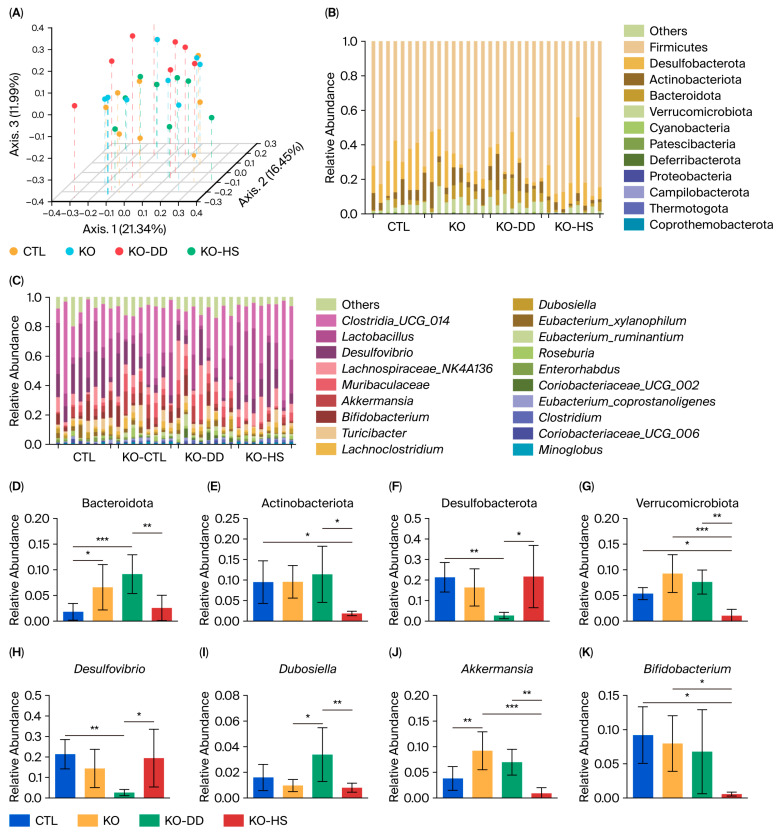
The effect of CVS treatment on gut microbiota composition and structure in *Apoe*^−/−^ mice. (**A**) PCoA plot. (**B**) Bacterial taxonomic profiling of the gut microbiota at the phylum level. (**C**) Bacterial taxonomic profiling of the gut microbiota at the genus level. The relative abundances of Bacteroidetes (**D**), Actinobacteriota (**E**), Desulfobacterota (**F**), Verrucomicrobiota (**G**), *Desulfovibrio* (**H**), *Dubosiella* (**I**), *Akkermansia* (**J**), and *Bifidobacterium* (**K**). Data presented as mean ± SD (n = 8). * *p* < 0.05, significant difference; ** *p* < 0.01, highly significant difference; *** *p* < 0.001 extremely significant difference.

The 16S rRNA sequencing was used to analyze the gut microbiota of mice. The results of α diversity showed significant differences between the two CVS treatment groups (Figure S1). The results of principal coordinate analysis (PCoA) based on Bray–Curtis dissimilarity showed that the CTL and KO samples partially overlapped. The KO-HS group was closer to the CTL group, and the KO-DD group was more inclined toward the new direction, rather than shifting it to a level similar to the CTL group (Figure 2A). These results indicate that DD-CVS regulates the gut microbial structure, which may be different from that of HS-CVS. The two CVS treatments significantly altered the composition of the gut microbiota (Figure 2B,C). At the phylum level, the results showed that Firmicutes, Desulfobacterota, Bacteroidota, Actinobacteriota, and Verrucomicrobiota had significant changes (Figure 2B). The relative abundance of Actinobacteriota and Bacteroidota was significantly decreased in the KO-HS group compared with the KO-DD group (Figure 2D,E). Meanwhile, compared with the CTL group, the relative abundance of Desulfobacterota significantly decreased in the KO-DD group (Figure 2F). The relative abundance of Verrucomicrobiota was significantly decreased in the KO-HS group (Figure 2G).

At the genus level, *Bifidobacterium*, *Dubosiella*, *Desulfovibrio*, *Akkermansia*, *Muribaculaceae*, and *Clostridia_UCG-014* had significant changes (Figure 2C). *Desulfovibrio* was significantly decreased in the KO-DD group, whereas *Dubosiella* was significantly increased (Figure 2H,I). The relative abundance of *Akkermansia* was significantly increased in the KO group compared with the CTL group, but it decreased in both CVS groups compared with the KO group (Figure 2J). The relative abundance of *Bifidobacterium* in the KO-HS group significantly decreased compared with the KO group. The relative abundance of *Bifidobacterium* in the gut of mice in the KO-DD group was significantly higher than that in the KO-HS group, a result consistent with the changes observed in the phylum Actinobacteria (Figure 2K). These results indicate that two CVS treatments could regulate gut homeostasis of *Apoe*^−/−^ mice, although the gut microbiota regulated by the two CVS types are not identical.

### 3.3. Effect of CVS on Microbial Biomarkers and Functions in Apoe^−/−^ Mice

**Figure 3:**
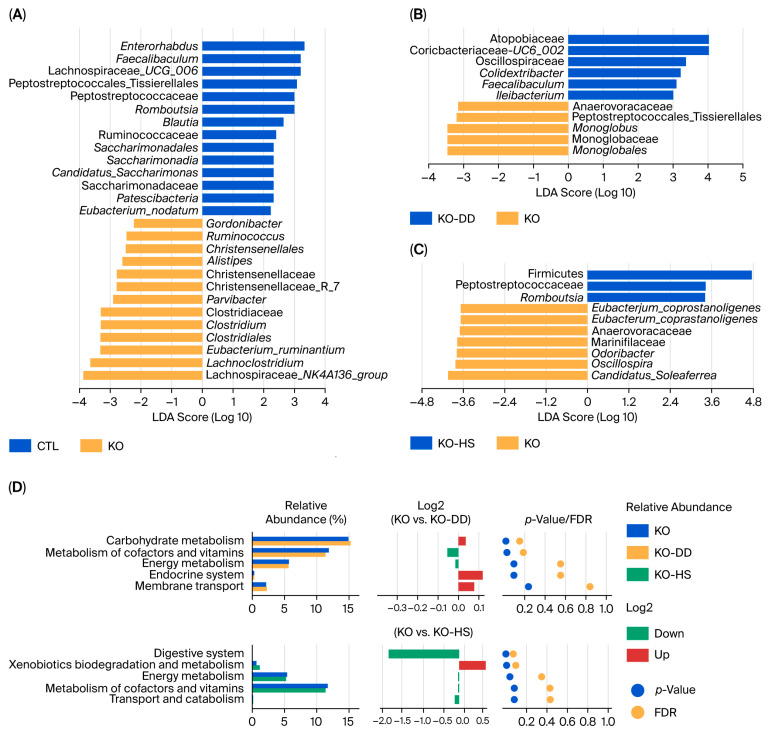
The effects of CVS treatment on differential gut microbiota and functional profiles of the gut microbiota in *Apoe*^−/−^ mice. (**A**–**C**) LEfSe analysis of gut microbiota was visualized by a distribution histogram based on the LDA score. (**D**) Differential functional profiles of the gut microbiota between the KO-DD group and the KO group, as well as the KO-HS group and the KO group.

To further confirm the biomarkers, we performed linear discriminant analysis (LDA). The result showed that the marker microbiota of the CTL group were *Enterorhabdus*, *Faecalibaculum*, *Romboutsia*, and *Blautia*. The marker microbiota in the KO group were *Lachnoclostridium*, *Eubacterium_ruminantium*, *Clostridiales*, and *Clostridium* (Figure 3A). Meanwhile, the marker microbiota of the KO-DD group were Clostridiaceae, Atopobiaceae, Oscillospiraceae, and Ruminococcaceae (Figure 3B). The significantly different microbiota in the KO-HS group were *Romboutsia*, *Odoribacter*, and *Oscillospira* (Figure 3C). These results show that CVS treatment leads to significant changes in gut differential microbiota, and different CVS treatments regulate different marker microbiota. In terms of the effect of CVS on the gut microbial function of mice, the function was predicted using PICRUST2 software (version 2.3). The results showed that the pathways predicted for the intestinal microbial function of mice in the KO-HS group included cofactor and vitamin metabolism, energy circulation, and other amino acid metabolism (Figure 3D). The pathways predicted in the KO-HS group included the digestive system, xenobiotics biodegradation and metabolism, and energy metabolism (Figure 3D). The above results indicate that organic acids in CVS may help digest and absorb nutrients in food, enhance energy metabolism, and reduce fat accumulation. However, it is worth noting that functional predictions were performed for exploratory purposes and may not fully reflect in vivo microbial functions.

### 3.4. Effect of CVS on Intestinal Metabolism of Apoe^−/−^ Mice

**Figure 4:**
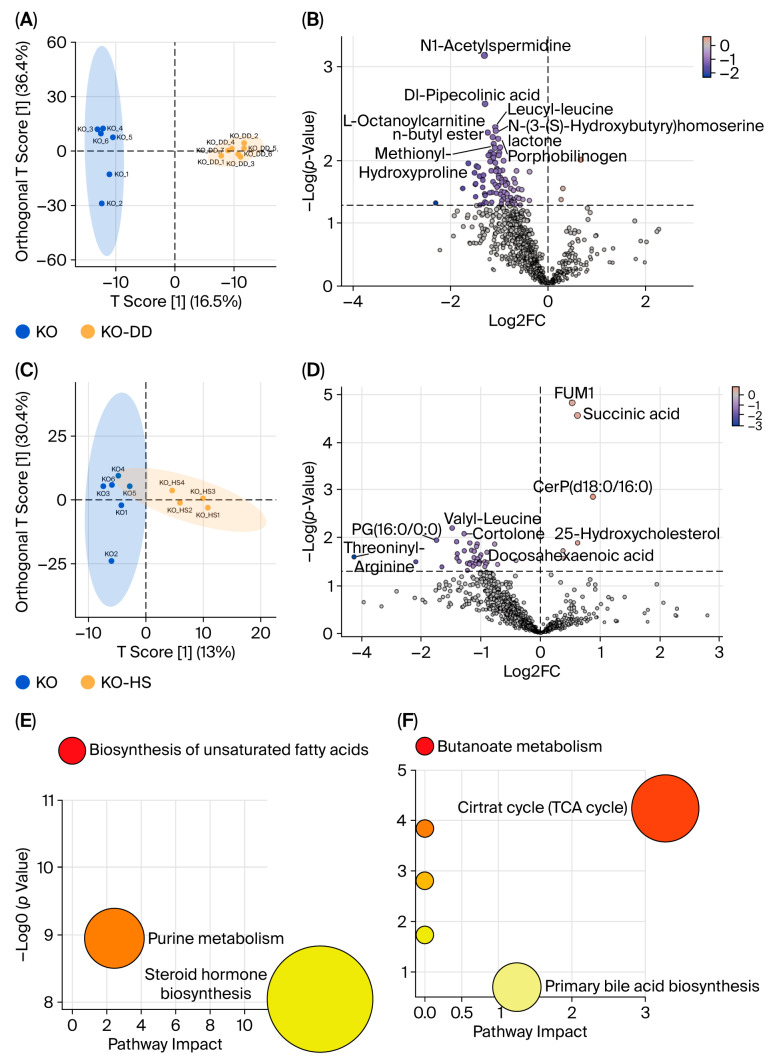
The effects of CVS treatment on metabolites of cecal contents in *Apoe*^−/−^ mice. The OPLS-DA of cecal metabolites, comparing KO-DD vs. KO (**A**) and KO-HS vs. KO (**C**). The volcano plots were visualized for differential metabolites for KO-DD vs. KO (**B**) and KO-HS vs. KO (**D**). KEGG pathway enrichment analysis for KO-DD vs. KO (**E**) and KO-HS vs. KO (**F**).

Based on the effect of CVS on gut microbiota, non-targeted metabolomics analysis was conducted on the microbial metabolites. In total, 816 metabolites were detected using LC-MS/MS under positive and negative modes (Table S2). The results of OPLS-DA showed that there was a clear separation between the KO group and KO-DD group or KO-HS group (Figure 4A,C). N1-Acetylspermidine, Dl-pipecolinic acid, leucyl-leucine, and methionyl-hydroxyproline in the cecal metabolites of the KO-DD group were significantly decreased (Figure 4B). HS-CVS treatment increased the level of succinic acid and 25-hydroxycholesterol and decreased the level of valylleucine, threoninylleucine, and cortolone (Figure 4D). These results indicate that the CVS intervention significantly affected the metabolic profile of microbiota. Furthermore, the KO-DD group exhibited microbial metabolite pathway enrichment in the biosynthesis of unsaturated fatty acids, purine metabolism, and steroid hormone biosynthesis (Figure 4E). The microbial metabolite pathways significantly enriched in the KO-HS group were the primary bile acid biosynthesis and citric acid cycle (Figure 4F). These results suggest that both CVS interventions regulate gut microbial metabolites and their metabolic pathways in hyperlipidemic mice.

### 3.5. Correlations Between Microbiota, Metabolites, and Physicochemical Indexes

**Figure 5:**
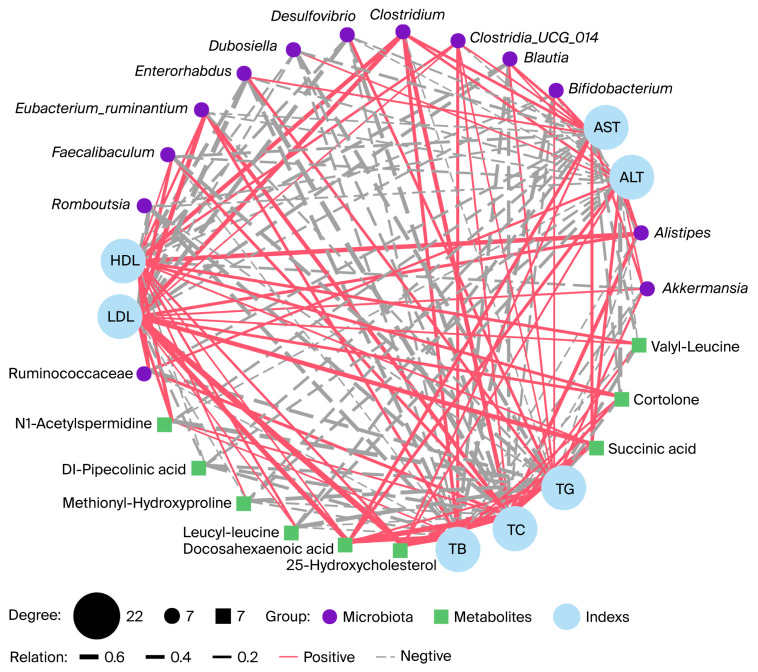
Gut microbiota and gut microbiota metabolites are associated with serum indices. The network diagram illustrates Spearman’s correlation analysis among the serum indices, gut microbiota, and gut microbiota metabolites in *Apoe*^−/−^ mice. The red solid line and the grey dashed line indicate positive correlation and negative correlation.

We further analyzed the correlations between key gut microbes and their metabolites with host parameters following CVS intervention (Figure 5). The results showed that *Enterorhabdus* was significantly negatively correlated with LDL and TC (r = −0.60, −0.59, *p* < 0.01), and *Romboutsia* showed similar negative correlations with TB and TC (r = −0.60, −0.59, *p* < 0.01). Although *Bifidobacterium* exhibited negative correlations with TB, TC, and ALT, these were not statistically significant. Notably, ALT levels were negatively correlated with cortolone (r = −0.47, *p* < 0.05), and TG levels were negatively correlated with N1-acetylspermidine (r = −0.52, *p* < 0.05). Levels of 25-hydroxycholesterol and succinic acid were strongly positively correlated with TC (r = 0.71, *p* < 0.001; r = 0.60, *p* < 0.01) and LDL (r = 0.76, *p* < 0.001; r = 0.52, *p* < 0.05), suggesting that changes in these metabolites may play a key role in regulating host TC levels. In addition, *Alistipes* was positively correlated with HDL (r = 0.53, *p* < 0.05). These results suggest that key gut microorganisms and metabolites exhibit significant associations with the indices of the host.

## 4. Discussion

CVS is a natural byproduct formed during the aging process of traditional grain vinegar fermentation. It has multiple pharmacological effects, such as anti-hyperlipidemia, anti-hyperglycemia, and liver protection. However, accumulating evidence suggests that food with health-beneficial properties may offer a complementary therapeutic strategy. This study explored the impact and potential mechanisms of CVS on hyperlipidemic mice through an integrated analysis of gut microbiome and metabolomic profiles.

We evaluated the biochemical characteristics of serum. This study demonstrates that CVS treatment had limited effects on the serological indicators of *Apoe*^−/−^ mice. The modest impact of CVS on serological markers such as TB, TC, and LDL may be attributed to the complexity of reversing the alterations caused by ApoE deficiency. Nonetheless, the significant reduction in TG levels and the increase in HDL in the KO-DD group suggest a potential beneficial modulation by CVS. Consequently, the associated pathological alterations are likely more complex and resistant to correction than those induced by acquired factors [17,18].

Furthermore, our findings reveal that DD-CVS and HS-CVS differentially reshape the gut microbial ecosystem in *Apoe*^−/−^ mice. The distinct clustering in the PCoA plot indicates that DD-CVS and HS-CVS drive the microbiota in different directions, implying potentially distinct mechanisms of action. The DD-CVS treatment increased the relative abundance of beneficial gut microbiota compared to HS-CVS treatment, such as commensal bacteria Bacteroidota [19]. At the genus level, DD-CVS promoted the enrichment of beneficial genera such as *Dubosiella*, which is known to strengthen the mucosal barrier via short-chain fatty acid production [20]. The relative abundance of *Bifidobacterium* and *Akkermansia* significantly increased in the KO-DD group compared with the KO-HS group. *Bifidobacteria* and *Akkermansia* are important genera within the phyla Verrucomicrobiota and Actinobacteriota, respectively. *Bifidobacterium*, a key commensal genus involved in host energy metabolism, may contribute to the regulation of lipid homeostasis and obesity-related phenotypes, potentially through modulating gut microbial composition and metabolite profiles [21,22,23]. The change in the relative abundance of *Akkermansia* is different from that suggested by previous research results [24]. The same type of bacteria can play opposite roles in different individuals and different environments. Grant et al. pointed out that *Akkermansia*, as a key member of the intestinal microbiota, exhibits dual characteristics of promoting metabolic health and potential pathogenicity, and its effects strongly depend on the environmental background [25]. Therefore, further research is needed to understand the complex functions of *Akkermansia*. Concurrently, DD-CVS reduced the abundance of opportunistic pathogens like *Desulfovibrio.* Studies have reported that *Desulfovibrio* is an opportunistic pathogen that may overgrow in various intestinal and extraintestinal diseases [26,27].

The LDA further confirmed that CVS treatments specifically altered key microbial biomarkers. The microbial biomarkers in the CTL group included *Enterorhabdus*, *Romboutsia*, and *Blautia*. The study has reported that *Enterorhabdus* may represent a potentially beneficial bacterium within the gut microbiota, and its significant enrichment has been shown to alleviate intestinal injury [28]. *Romboutsia* has also been identified as a probiotic; supplementation with *Romboutsia lituseburensis* JCM1404 was reported to modulate the gut microbiota and lipid metabolism in obese rats [29]. Moreover, Xu et al. demonstrated that the gut bacterium *Blautia* could effectively improve hyperlipidemia in mice [30], while another study found that a low-fat diet increased the abundance of *Blautia*, thereby contributing to host lipid metabolism [31]. The marker microbiota of the KO group were significantly different from the CTL group, including *Lachnoclostridium*, *Eubacterium_ruminantium*, *Clostridiales*, and *Clostridium*. *Lachnoclostridium* has been associated with obesity, hypercholesterolemia, and inflammation [32]. Several studies have suggested that a high abundance of *Lachnoclostridium* may reduce circulating levels of metabolites such as acetate and bile acids, thereby exerting adverse effects on obesity and hyperlipidemia [33,34]. Both CVS treatments specifically altered key microbial biomarkers. The KO-DD group was characterized by an increase in Ruminococcaceae, a family of short-chain-fatty-acid-producing bacteria associated with a healthy gut [35]. DD-CVS may regulate the relative abundance of Ruminococcaceae, altering gut microbial metabolism and thereby modulating the gut microbiota homeostasis in *Apoe*^−/−^ mice. The KO-HS group exhibited microbial biomarkers similar to those of the CTL group, indicating a restoration of gut microbiota composition consistent with the β-diversity findings. Moreover, our study has confirmed that changes in Oscillospiraceae are closely associated with obesity, meaning that they may be a candidate for probiotics with the effects of weight loss, lipid reduction, and relief of metabolic syndrome [36].

The results of gut microbial function prediction showed that the pathways predicted for the DD-CVS treatment included cofactor and vitamin metabolism, energy circulation, and other amino acid metabolism. Additionally, the pathways predicted by HS-CVS treatment included the digestive system, xenobiotic biodegradation and metabolism, and energy metabolism. This finding is consistent with the results of gut microbiota analysis. Commensal bacteria such as Bacteroidota play a crucial role in energy harvest and metabolism in the gut, and changes in their abundance may contribute to the observed improvements in microbial metabolism. A previous study reported that supplementation with adzuki beans has been shown to modulate the amino acid metabolism of the gut microbiota in mice, thereby alleviating obesity [37]. However, this study acknowledges that functional predictions based on PICRUSt2 may only provide a preliminary reference for potential microbial functions. These predictions do not directly reflect gene expression or enzymatic activity and therefore may not fully capture the actual functional state in vivo. Consequently, the results derived from PICRUSt2 should be interpreted activity and further experimental validation is warranted to confirm the predicted functions.

At the metabolomic level, the reduction in N1-acetylspermidine by DD-CVS treatment could be beneficial, given its reported role in promoting immune suppression mediated by macrophages, which may consequently affect the efficacy of immunotherapy [38]. The increase in succinic acid with HS-CVS treatment is particularly interesting, as this metabolite has recently been shown to promote white adipose tissue browning and improve fat metabolism [39]. However, evidence indicates that succinic acid displays a dual nature in its biological functions. Accumulating evidence suggests that increased succinate levels are associated with the development and progression of multiple diseases, such as MASH, fibrosis, and liver cancer [40,41]. Both inflammation and malignancies are characterized by hypoxic microenvironments, where succinate accumulation may promote tumorigenic processes in the context of inflammation [42]. Therefore, succinate may be defined as a molecule that signals danger. In addition, a simultaneous increase in 25-hydroxycholesterol was observed. Moreover, 25-hydroxycholesterol exerts dual and complex roles in cholesterol homeostasis and immune regulation, which warrant further investigation. The study has reported that 25-hydroxycholesterol inhibits LDL uptake through suppression of the SCAP/SREBP-2 pathway. However, a high intracellular concentration of cholesterol increases 25-hydroxycholesterol production [43]. It has been reported that the accumulation of 25-hydroxycholesterol in macrophages induces the expression of arginase1, thereby creating a protumorigenic microenvironment that accelerates tumor progression [44].

In addition, CVS treatment altered microbial-metabolite-associated pathways in the gut. KEGG enrichment analysis revealed that the KO-DD group was significantly enriched in microbial pathways related to unsaturated fatty acid biosynthesis, purine metabolism, and steroid hormone biosynthesis. The KO-HS group exhibited significant microbial metabolite pathway enrichment in primary bile acid biosynthesis and the citric acid cycle. These pathways are closely associated with microbial energy metabolism and lipid regulation. Notably, bile acids act as key signaling molecules, and their microbial metabolism has been shown to modulate lipid homeostasis, hepatobiliary function, and gut microbial composition, thereby maintaining gut health [45].

The correlation analysis integrates these findings by directly linking specific microbes and metabolites to host parameters. The significant negative correlations between beneficial genera such as *Enterorhabdus* and *Romboutsia* with TC and LDL underscore their potential protective role. The positive correlation between *Alistipes* and HDL may support the notion that CVS-induced microbial changes are relevant to host lipid metabolism. Among the gut microbial metabolites, 25-hydroxycholesterol and succinic acid showed a positive correlation with TC and LDL levels. There is evidence suggesting that 25-hydroxycholesterol and succinic acid are beneficial for the host, due to their anti-oxidant and anti-inflammatory properties and enhancement of insulin sensitivity [42,43]. However, these metabolites are known to play complex and context-dependent roles in metabolic regulation and have also been described as danger-signaling molecules [41,46]. For instance, succinic acid has been reported to inhibit fatty acid oxidation via the AMPK/PPARα/FGF21 pathway, thereby promoting hepatic steatosis in non-obese NAFLD mice [47]. Another study showed that succinic acid induces gut microbiota dysbiosis and macrophage-mediated inflammatory lipid production by driving the gut–fat axis, leading to abdominal fat accumulation [48]. This evidence may explain the observed positive correlation between 25-hydroxycholesterol and succinic acid with TC and LDL in our study. It is important to note that CVS treatment improves gut microbiota composition and metabolic profiles in hyperlipidemic mice, and these key microbial taxa and metabolites were significantly correlated with host physiological indicators. However, the precise molecular mechanisms through which gut microbiota or their metabolites regulate hyperlipidemia need further research.

## 5. Conclusions

In this study, we investigated the effects of CVS on hyperlipidemia in *Apoe*^−/−^ mice from the perspectives of serum biochemistry, gut microbiota, and microbial metabolism. CVS supplementation showed limited improvement in serological indicators. Importantly, both CVS interventions significantly influenced the gut microbial composition and diversity, yet they exhibited distinct modulatory effects on specific taxa. In addition, metabolomic and functional enrichment analyses revealed that both CVS interventions can effectively and differentially reshape microbial metabolic profiles and metabolic pathways. Correlation analyses further highlighted that key metabolites and microbiota, such as N1-acetylspermidine, succinic acid, 25-hydroxycholesterol, *Enterorhabdus*, *Romboutsia*, and *Blautia*, were strongly associated with serum parameters. These results suggest that CVS primarily exerts its effects via modulation of the gut microbiota–metabolite axis in hyperlipidemic mice.

Taken together, our findings demonstrate that both CVS treatments robustly altered the gut microbiota–metabolite axis, with modest improvement in TG and HDL, while TC and LDL remained unchanged in *Apoe*^−/−^ mice. CVS treatment has limited effects in reversing hyperlipidemia in mice, which may be attributable to the complexity of genetically induced metabolic disorders and the short intervention period. The findings of this study provide a reference for research on the health functions of CVS.

## Data Availability

Data generated in this study can be obtained from the corresponding author upon reasonable request. The original data of 16S rRNA gene sequencing are available from the NCBI database (PRJNA1293934).

## Supplementary Materials

The following supporting information can be downloaded at: https://www.mdpi.com/article/10.3390/foods15030427/s1. Figure S1: Effect of CVS treatment on gut microbiota structure evaluated by α-diversity; Table S1. The basic chemical parameters in CVS^1^; Table S2. The information of metabolites for cecal contents under positive and negative mode.

## Abbreviations

CVS	Cereal vinegar sediment
DD-CVS	Dade-cereal vinegar sediment
HS-CVS	Hengshun-cereal vinegar sediment
KO	*Apoe* ^−/−^
CTL	control
KO-DD	Dade-cereal vinegar sediment treatment
KO-HS	Hengshun-cereal vinegar sediment treatment
*Apoe* ^−/−^	Apolipoprotein E–deficient
AST	aspartate aminotransferase
ALT	alanine aminotransferase
TC	total cholesterol
TB	total bilirubin
HDL	high-density lipoprotein
LDL	low-density lipoprotein
PLS-DA	partial least squares discriminant analysis
LDA	linear discriminant analysis
FC	fold change
