# Supplementary material for: Cereal Vinegar Sediment Modulates the Gut Microbiota–Metabolite Axis Associated with Hyperlipidemia in Apoe−/− Mice
Source: Foods. 2026 Jan 24;15(3):427. doi: 10.3390/foods15030427 (PMC12896822; doi:10.3390/foods15030427)
Supplement: Supplementary file 1 [file foods-15-00427-s001.zip › foods-4059507-supplementary.pdf]

Table S1. The basic chemical parameters in CVS[1]

Physicochemical Parameters	HS-CVS	DD-CVS
Moisture (%)	48.33 ± 0.00	65.00 ± 0.00
Total Acidity (g/100 mL)	6.87 ± 0.00	11.21 ± 0.36
Carbohydrates (mg/g)	46.85 ± 2.375	232.30 ± 0.00
Reducing Sugar (μg/g)	37.23 ± 0.00	116.90 ± 0.03
Total phenolic content (mg GAE/g)	4.73 ± 0.09	4.00 ± 0.06
Total flavonoid content (mg RE/g)	3.45 ± 0.03	2.60 ± 0.00
Proteins (g/100g)	3.08 ± 0.00	7.80 ± 0.09

[1] Data in the table represent mean ± S.D. (n = 3).

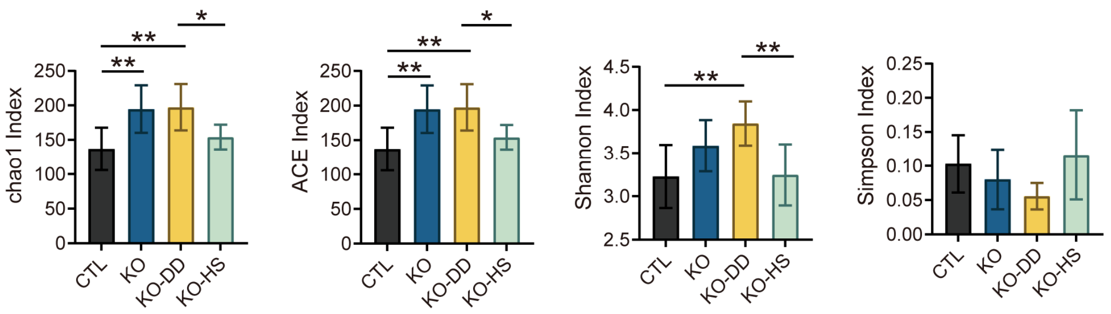

Figure S1. Effect of CVS treatment on gut microbiota structure evaluated by α-diversity. (A) Chao1 index, (B) ACE index, (C) Shannon index, and (D) Simpson index.
